# The Importance of Coping and Emotion Regulation in the Occurrence of Suicidal Behavior

**DOI:** 10.1177/0033294118781855

**Published:** 2018-06-21

**Authors:** Elsie Ong, Catherine Thompson

**Affiliations:** School of Health Sciences, University of Salford, UK

**Keywords:** Emotion regulation, coping, suicidal behavior, cognitive reappraisal, expressive suppression

## Abstract

Research has shown that the use of maladaptive coping strategies and difficulties in regulating mood are linked to increasing risk of suicide. This study measured the impact of coping and emotion regulation on suicidal behavior in a sample of Asian students. The aim was to determine whether different coping strategies and methods of expressive suppression and cognitive reappraisal would be associated with suicidal behavior. One hundred and twenty undergraduate students were recruited from The Open University in Hong Kong and all completed questionnaires that measured coping, emotional regulation, and suicidal behavior. The results showed that increased avoidance coping was associated with increased suicidal behavior, whereas increased cognitive reappraisal was associated with reduced risk of suicidal behavior. Specifically, in an Asian student population, avoidance coping appears to be a risk factor for suicide, while cognitive reappraisal may be seen as a positive, protecting strategy.

## Introduction

Suicide is not an isolated process, but rather is conceptualized as a continuum of processes starting with suicide ideation (SI; the recurrent thoughts of committing suicide), moving to the formation of a suicide plan, a suicide attempt, and then finally suicide completion (Lewinsohn, Rohde, & Seeley, 1996). These stages are all referred to as aspects of suicidal behavior, and to prevent suicide at an early point, it is important to identify the risk factors that lead an individual to engage in SI and then continue through the suicide path.

Although researchers have established risk factors associated with suicide including depression, poor coping abilities, higher avoidance of stressors, and a lack of close social relationships (John & Gross, 2004), the work has had limited success in predicting and preventing suicide based on the identification of these risk factors. This is because many individuals experience similar negative situations but not all will consider suicide (Bazrafshan, Jahangir, Mansouri, & Kashfi, 2014). Therefore, it is postulated that when faced with a situation, some individuals are more motivated to proceed to a suicide act, while others are more resilient and will not engage in suicidal behaviors. This raises the question as to whether some individuals may have specific capabilities that make them less prone to suicidal behavior.

### Emotion regulation and suicidal behavior

Rajappa, Gallagher, and Miranda (2011) examined how suicidal behavior was linked to difficulties in managing negative mood (also referred to as emotion regulation). Emotion regulation is a set of regulatory processes that can be used to redirect emotions in order to modify the magnitude, latency, and duration of affective responses (Miranda, Gaudreau, Debrosse, Morizot, & Kirmayer, 2012; Thompson, 1994). It includes the management of both positive and negative emotions that arise under a wide range of stressful and non-stressful situations (Compas, Connor-Smith, Saltzman, Thomsen, & Wadsworth, 2001). Rajappa et al. (2011) studied the links between emotion regulation and suicide by measuring different emotion regulation strategies (e.g., awareness, clarity, non-acceptance, impulse, goals, and strategies) among young adults with varying experience of suicidal behavior. The sample included 17 adults who reported current SI, 20 with one past suicide attempt, 17 with multiple suicide attempts, and 42 control participants with no history of suicide. Results showed that failure to adopt these emotion regulation strategies and non-acceptance of emotional responses significantly predicted SI. Rajappa et al. (2011) suggest that suicidal behaviors are attempts to escape negative emotions and they present when individuals lack emotion regulation strategies in response to emotional distress. The finding shows the importance of emotion regulation in suicidal behavior.

The process model of emotion regulation (Gross & John, 1998) outlines two emotion regulation strategies, cognitive reappraisal and expressive suppression. Cognitive reappraisal is defined as the attempt to reinterpret an emotion-eliciting situation in a way that alters the meaning and changes the emotional impact (Gross & John, 2003). Cognitive reappraisal aims to reduce negative emotions by changing the interpretation or appraisal of affective stimuli. For example, an individual experiencing anxiety at a job interview may try to reframe the stressful situation as a learning experience. In contrast, expressive suppression is an attempt to hide, inhibit, or reduce on-going emotion-expressive behavior. For example, an individual may try to disguise their anxiety at a job interview by breathing slowly and trying to appear confident. Cognitive reappraisal is employed before an emotional response has been fully generated or activated (Gross & Thompson, 2007; Ochsner, et al., 2004; Ochsner, et al., 2004) and is focused on altering the effect of emotion-generating cues. It is referred to as an antecedent-focused strategy (Gross, 1998). Expressive suppression is a response-focused strategy that acts later in the emotion generation process and attempts to modify the behavioral expression of the emotion after it has been experienced (Gross, 2002; Gross & John, 2003).

### The importance of cognitive reappraisal and expressive suppression

Cognitive reappraisal is considered to be a more effective method to regulate emotion and physiological arousal, because it requires fewer cognitive and physiological resources compared to expressive suppression (Ochsner, Silvers, & Buhle, 2012). In addition, because cognitive reappraisal occurs before the complete activation of emotion response tendencies has taken place, regulating emotion in this way does not create a discrepancy between inner experience and outer expression that is experienced in expressive suppression. Individuals who frequently use expressive suppression are less aware of their own feelings and often experience disruptions in social relationships (Butler et al., 2003; Gross & John, 2003). It is argued that this strategy is accompanied by a resistance to seek and receive help, and consequently, individuals who engage in expressive suppression tend to have less social support, poor coping abilities, higher avoidance of social situations, and a lack of close social relationships. All of these factors are known to increase the risk of suicidal behavior (John & Gross, 2004).

In contrast to the negative impact of expressive suppression, Gross and John (2003) have found that those who primarily engage in cognitive reappraisal are more likely to share their emotions (both positive and negative) with others and maintain close relationships with friends. They also use more self-regulation strategies in the form of coping and have a stronger social network compared to expressive suppressors. Coping involves both cognitive and behavioral efforts aimed at reducing or controlling a stressor (Stone, Helder, & Schneider, 1988, p. 183). Coping is associated with maximizing the experience of positive emotions, reducing the impact of negative emotions, and enabling adaptive outcomes in individuals (Bonanno & Burton, 2013; Carlson et al., 2015; Sheppes et al., 2014). Although coping is sometimes conceived as a special category of emotion regulation under the presence of stress (e.g., Eisenberg et al., 2007), it is primarily directed at decreasing negative affect to a stressor (Compas et al., 2013; Lazarus & Folkman, 1984). In contrast, emotion regulation can be applied to a wider range of circumstances (stressful and non-stressful) than coping (e.g., Gross & Thompson, 2007). For example, emotion regulation may involve the management of positive emotional responses such as suppressing a laugh when seeing someone trip over on the street, whereas coping is exclusively limited to responses to stressors (Compas et al., 2013). In addition, coping is generally considered to be longer lasting and is often applied to stressors such as bereavement or diagnosis of a chronic condition (Carver, Scheier, & Weintraub, 1989). In contrast, emotion regulation usually occurs over a relatively short time frame and varies considerably with different goals and situations (Gross & John, 2003).

In a series of five studies, Gross and John (2003) investigated individual differences in the use of suppression and reappraisal among undergraduate students (N = 1483). All participants were asked to complete self-report measures of depression (indexed by the Beck Depression Inventory; Beck, Steer, & Brown, 1996), positive wellbeing (using the Ryff Scales of Psychological Well-Being; Ryff & Keyes, 1995), and emotion regulation (using the Emotion Regulation Questionnaire [ERQ]; Gross & John, 2003). They found that the use of expressive suppression was associated with lower levels of positive wellbeing and higher levels of depression. It was suggested that when participants try to suppress negative emotions, they mask their inner feelings and minimize outward displays of emotion. Consequently, these individuals are less honest about how they are feeling, they are less effective in regulating their negative emotions, and they view their emotions in a less favorable or accepting way (as indicated by low scores on the subscale of “self-acceptance” in the wellbeing measure). Contrary to the use of expressive suppression, individuals who used cognitive reappraisal showed improved wellbeing and reported fewer negative emotions than those who reappraised less frequently. Consequently, individuals who use cognitive reappraisal often experience more positive emotions and fewer negative emotions than those who use this strategy less often.

The benefits of using cognitive reappraisal in preventing suicide have also been found in more recent studies. Using self-report measures of the ERQ, Richmond, Hasking, and Meaney (2017) found that while depression, anxiety, and stress each exerted a direct effect on non-suicidal self-injury among 1586 university students, the increased use of cognitive reappraisal had a greater impact on reducing suicide risk than the use of expressive suppression. Similarly, Forkmann et al. (2014) found that increased use of expressive suppression and reduced use of cognitive reappraisal significantly predicted increased SI. These results highlighted possible interventions for students experiencing psychological distress such as having workshops or training on the use of adaptive emotion regulation strategies.

### The importance of adaptive coping

In addition to emotion regulation, research has shown that the use of coping strategies also has an impact on suicidal behavior, and both cognitive and behavioral aspects of managing emotions are important in an individual’s resilience against suicide (Görgen, Joormann, Hiller, & Witthöft, 2015; Zhang, Wang, Xia, Liu, & Jung, 2012). Coping is often categorized into adaptive and maladaptive strategies. For example, Zuckerman and Gagne (2003) propose that adaptive coping consists of approach, self-help, and accommodation coping and maladaptive coping consists of avoidance and self-punishment. Past findings generally support the notion that adaptive coping strategies are more effective in difficult situations and when managing negative emotions than maladaptive strategies. For instance, higher scores on measures of self-punishment and avoidance coping (maladaptive) are positively associated with SI in veterans (Pietrzak, Russo, Ling, & Southwick, 2011) and police officers (Pienaar, Rothmann, & van de Vijer, 2007). In contrast, lower scores on measures of approach coping (adaptive) are negatively associated with SI (Pienaar et al., 2007).

Richard-Devantoy, Yang, Gustavo, & Fabrice, 2017) argue for the importance of exploring coping and emotion regulation in suicidal behavior, because they found that individuals are at greater risk of attempting suicide when they lack adaptive coping skills and are less able to regulate their emotions and emotional responses. However, because the constructs of coping and emotion regulation are similar in many ways, the independent study of each can be difficult.

### Cross-cultural differences in coping and emotion regulation

Despite the fact that coping and emotion regulation are both important in suicide, it is noteworthy that there are significant cultural variations in coping and emotion regulation and these should be accounted for. To date, much of the past research has been conducted in the West with little understanding of how the findings extrapolate to an East Asian sample (Wong, Wong, & Scott, 2006). It is suggested that the emotion regulation strategies most often used by individuals in the West may not be used by those living in East Asian cultures, for example, the extensive use of cognitive reappraisal but reduced use of suppression in the studies conducted by Gross and John (2003) and Richard-Devantoy et al. (2017). An example of an East Asian culture is Hong Kong and Yuan, Liu, Ding, and Yang (2014) proposed that Hong Kong is immersed in Chinese culture that is characterized by collectivistic cultural norms with relational harmony and self-discipline. To some extent, individuals are culturally trained to suppress the expression of negative emotions. Therefore, it may be argued that suppression may be an adaptive and effective emotion regulation strategy in East Asian cultures. This suggestion is consistent with studies that have found expressive suppression to be culture-specific. For instance, research shows that the use of suppression may relate to fewer negative emotional experiences, improved social interactions, and more positive physiological responses in individuals with East Asian cultural values (Butler, Lee, & Gross, 2007; Mauss & Butler, 2010; Soto, Perez, Kim, Lee, & Minnick, 2011).

The fact that the use of coping and emotion regulation may differ cross-culturally and the focus of past research on Western cultures means it is important to explore the impact of these factors in East Asian cultures. In contrast to suicide being the 10th leading cause of death for all age groups (see the Center for Disease Control and Prevention, 2015), it is the second leading cause of death for young people aged 15 to 24 years. The high numbers of suicide in this age group is an even bigger concern in the Chinese culture as suicide is the leading cause of death among adults aged 15 to 34 in China and accounts for 19% of deaths in this age group (Phillips, Li, & Zhang, 2002). This again highlights the importance of studying risk factors associated with suicide in this population.

In addition to the high rates of suicide in young people, findings also show that those attending college or university report more SI than their non-college peers (Mortier, et al., 2018). In the academic year 2015–2016, a total of 22 young people in Hong Kong committed suicide within a six-month period, and 10 of these were university students (South China Morning Post, 2016). This contributed to a suicide rate of 8.3 per 100,000 in 2016 which is a relatively high figure compared to previous figures of 6.2 per 100,000 in 2014. This indicates a concerning increase in suicide in this population (see the University of Hong Kong’s Centre for Suicide Research and Prevention 2010 to 2014). Pereira and Cardoso (2015) explained that this may be due to the critical transition students go through during their academic life, which in turn exposes them to more stress and challenges (compared to their non-student peers). Therefore, it is crucial that university students have adaptive ways to cope with the challenges they are exposed to as poor coping and emotional regulation strategies may be detrimental to their development and progress.

### Aims and intentions of the current study

The present study explored the relationship between emotion regulation, coping, and suicidal behavior in a non-clinical student population from Hong Kong. The intention of the work was to investigate the effects of coping and emotion regulation on a group who are at risk of suicidal behavior, but showing relatively minimal signs of this behavior. This will help to determine whether coping and emotion regulation could be used to identify and prevent suicidal behavior before it reaches a critical point and leads to a suicide attempt. By studying coping and emotion regulation in a population from Hong Kong, the work will determine whether cultural differences have an impact on the use of coping strategies and whether this has a different impact on resilience to suicide in comparison to past studies that focus on Western populations.

Participants were asked to complete three self-report measures of suicidal behavior, coping, and emotion regulation. It was predicted that increased use of emotional suppression and maladaptive coping strategies would be associated with increased suicidal behavior, whereas the use of cognitive reappraisal and adaptive coping would be associated with reduced suicidal behavior.

## Method

### Design

This investigation was a correlational study to explore the relationship between suicidal behavior and strategies of coping and emotion regulation. The variable of suicidal behavior gave a measure of the extent to which participants were experiencing SI and took account of past suicide attempts and thoughts. Coping was measured on five dimensions of self-help, approach, accommodation, self-punishment, and avoidance. The variable of emotion regulation was separated into cognitive reappraisal and expressive suppression. The study was approved by the Ethics Committee of the School of Health Sciences at the University of Salford.

### Participants

One hundred and twenty undergraduate students from The Open University in Hong Kong (51 males, 69 females) were recruited for this study using convenience sampling. Their ages ranged from 18 to 28 years, with a mean of 23.14 years (SD = 5.51). Prospective participants were prescreened for any history of neurological and cognitive deficits (based on the findings of Richard-Devantoy et al., 2017). Three participants were excluded leaving a sample size of 117 participants.

### Materials

Three questionnaires were used for this study in order to measure each of the variables. All were presented in English. Suicidal behavior was measured using the Suicidal Behaviours Questionnaire–Revised (SBQ-R; Osman et al., 2001). This is a 4-item inventory that measures past, current, and future suicidal thoughts and attempts. Item 1 measures lifetime SI and/or suicide attempts, item 2 assesses the frequency of SI in the previous 12 months, item 3 quantifies the threat of a suicide attempt, and item 4 is the self-reported likelihood of future suicidal behavior. Each question is answered using a Likert scale and the scale for each question differ slightly. The scales range from a minimum of 0 to a maximum of 6, with lower numbers indicating a relatively low risk of suicide. Total scores on the questionnaire range from 3 to 18 and represent overall suicide risk whereby higher scores represent greater risk. Scores of 7 or above indicate significant risk of suicidal behavior. Cronbach's alpha for the SBQ-R in an undergraduate student population ranges from 0.76 (Osman et al., 2001) to 0.80 (Aloba, Ojeleye, & Aloba, 2017).

Emotion regulation was measured using the ERQ (Gross & John, 2003). This is a 10-item questionnaire measuring individual differences in respondents’ habitual use of two strategies for regulating their emotions, cognitive reappraisal (six items), and expressive suppression (four items). Each question is rated on a seven-point Likert scale (from 1 = “strongly disagree” to 7 = “strongly agree”) and a higher score indicates greater use of a particular strategy. The range of scores for reappraisal is 6 to 42, and for suppression is 4 to 28. Internal reliability for the ERQ in a student population is acceptable with Cronbach's alpha reported as 0.82 for cognitive reappraisal and 0.76 for expressive suppression (Gross & John, 2003).

Coping strategies were measured using the revised COPE (R-COPE; Zuckerman & Gagne, 2003). This is a 40-item inventory that assesses five forms of coping in response to stress: self-help, approach, accommodation, avoidance, and self-punishment. S*elf-help coping* signifies seeking support and dealing with an incident by understanding and expressing one’s own emotions. *Approach coping* focuses on problem solving and *accommodation coping* measures the ability to accept that a problem cannot be resolved and the willingness to use positive reframing to develop an optimistic outlook of the incident. These three strategies of coping are collectively called *adaptive coping* as they relate to positive mental outcomes. The remaining two coping strategies, *avoidance* and *self-punishment* are grouped as *maladaptive coping* strategies*.* Avoidance coping aims to direct the individual away from a problem via disengagement, denial, and blaming external forces for the situation. Self-punishment measures maladaptive coping strategies such as self-focused rumination and self-blame regardless of whether these contributed to the incident (Zuckerman & Gagne, 2003).

The R-COPE has been modified from the classic coping inventory (COPE; Carver et al., 1989). Each question is answered using a four-point scale (from 1 = “I usually don’t do this at all” to 4 = “I usually do this a lot”). Each dimension of coping is measured using eight questions, giving a minimum score of 8 and a maximum of 32, with higher scores indicating greater use of a particular strategy. The R-COPE has reported high internal validity with a Cronbach’s alpha ranging from 0.81 to 0.92 (Zuckerman & Gagne, 2003).

### Procedure

All participants were given an information sheet and consent form together with the questionnaires. All materials were distributed to students during a lecture and they were asked to read the instructions and then individually complete each questionnaire at their own pace. The completion of the questionnaires took approximately 30 min, and following completion participants were debriefed by the researcher.

## Results

Results included scores on the SBQ-R, the scores for the two emotion regulation strategies, and scores for the five dimensions of coping. The descriptive statistics for each measure can be found in Table 1. There were 70 participants (59.83%) who scored below 7 on the SBQ-R (median = 4.00, range = 3–6), and a total of 47 participants (40.17%) who scored higher than 7 (indicating a relatively high suicide risk (median = 9.00, range = 7–15). Five participants (4.24%) reported at least one past suicide attempt. A series of Spearman’s correlations were conducted to analyze the relationship between suicidal behavior and the different strategies of coping and emotion regulation (see Table 2). Out of the five coping strategies, suicidal behavior was positively correlated with avoidance coping, *r*_(117)_ = .25, *p* < .01, indicating that higher levels of suicidal behavior were related to increased use of avoidance coping. Suicidal behavior was negatively correlated with accommodation coping, *r*_(117)_ = −.20, *p* < .05, and self-help coping, *r*_(117)_ = −.23, *p* < .05. This shows that participants who reported less use of accommodation and self-help coping showed higher levels of suicidal behavior. There was no significant correlation between suicidal behavior and self-punishment, *r*_(117)_ = .17, *p* > .05, or approach coping, *r*_(117)_ = −.09, *p* > .05.

**Table 1.** Descriptive statistics for the study variables.

	Median	Range	Mean	SD	α
Suicidal behavior	5.5	3–15	5.97	3.21	0.761
Cognitive reappraisal	27	10–40	27.26	5.58	0.813
Expressive suppression	15	7–24	15.98	3.62	0.619
Avoidance coping	17	9–28	16.97	4.33	0.645
Self-punishment coping	19	9–31	19.29	5.08	0.770
Accommodation coping	21	12–32	21.27	4.55	0.682
Approach coping	23	15–35	20.50	4.71	0.717
Self-help coping	20	9–32	20.55	5.60	0.834

SD: standard deviation; α: Cronbach’s alpha.

**Table 2.** Correlations between suicidal behavior, emotion regulation, and coping.

	1	2	3	4	5	6	7	8
1. Suicidal behavior	1.00							
2. Cognitive reappraisal	−.34**	1.00						
3. Expressive suppression	.001	.13	1.00					
4. Avoidance coping	.25**	.00	.26**	1.00				
5. Self-punishment coping	.17	−.05	.24**	.44**	1.00			
6. Accommodation coping	−.20*	.26**	−.12	−.05	.01	1.00		
7. Approach coping	−.09	.23*	.01	.16	.33**	.45**	1.00	
8. Self-help coping	−.23*	.21*	−.42**	−.00	−.03	.36**	.31**	1.00

*Correlation is significant at the .05 level.

**Correlation is significant at the .01 level.

There was a negative correlation between scores on the SBQ-R and cognitive appraisal, *r*_(117)_ = −.34, *p* < .01, with increased use of cognitive reappraisal relating to reduced suicidal behavior. However, there was no relationship between suicidal behavior and expressive suppression, *r*_(117)_ = .01, *p* > .05.

To assess the impact of cognitive reappraisal, avoidance, accommodation, and self-help coping on suicidal behavior, a multiple regression analysis was performed with scores on the SBQ-R as the dependent variable and the different coping and emotion regulation strategies as predictor variables (Table 3). Using the enter method, a significant model emerged, *F*_(4,114)_ = 10.77, *p* < .001, adjusted R^2 ^= 0.255. The variables that were shown to significantly predict suicidal behavior were avoidance coping (Beta = 0.275, *p* < .001) and cognitive reappraisal (Beta = −0.343, *p* < .001). While they were associated with scores on the SBQ-R, accommodation coping (Beta = − 0.102, *p* = .28) and self-help coping (Beta = −0.148, *p* = .11) did not predict suicidal behavior.

**Table 3.** Multiple-regression to explore the extent to which the aspects of emotion regulation and coping can predict suicidal behavior.

Predictors	*b*	SE (B)	*t*
Cognitive reappraisal	−0.179	−0.343	−4.058**
Avoidance coping	0.214	0.275	3.366**
Accommodation coping	−0.076	−0.102	−1.097
Self-help coping	−0.085	−0.148	−1.611

**p < .001.

## Discussion

The aim of the present study was to investigate whether the use of specific coping and emotion regulation strategies could statistically predict suicidal behavior in a non-clinical sample of Asian students. This population has a high incidence of suicide, and currently, the findings in this field focus primarily on Western samples. Findings show cross-cultural differences in coping and emotion regulation strategies, and this study was unique in exploring risk factors associated with suicide in an East Asian sample. Identifying the risk factors and predictors in a non-clinical sample may also help to identify those at risk at a relatively early stage in the suicide process, therefore reducing suicidal behavior before it becomes more extreme.

Based on the existing literature regarding the link between emotion regulation strategies and suicidal behavior (Rajappa et al., 2011; Richard-Devantoy et al., 2017; Richmond et al., 2017; Sheppes et al., 2014), it was predicted that higher levels of expressive suppression and lower levels of cognitive reappraisal would be associated with increased risk of suicidal behavior. As hypothesized, the results showed that increased use of cognitive reappraisal was related to reduced suicidal behavior; however, there was no relationship between suicide and expressive suppression. This partially supports the majority of past findings which indicate that limited use of cognitive reappraisal is related to increased symptoms of mental health disorders and suicide risk (Campbell-Sills, Barlow, Brown, & Hofmann, 2006a; Garnefski & Kraaij, 2006; Gross & John, 2003; Joormann, Siemar, & Gotlib, 2007; Moore, Zoellner, & Mollenholt, 2008). The results support the argument that the ability to regulate emotion by reinterpreting an emotion-eliciting situation is a beneficial way of coping with stressful situations. One possible explanation for this is that individuals who use appraisal are less likely to assess a difficult situation as defeating or feel entrapped due to the situation. They may be more likely to seek alternative ways to cope by means of interpersonal problem solving and strengthening social support (Johnson, Gooding, & Tarrier, 2008; Johnson, Gooding, Wood, & Tarrier, 2010).

In relation to the process model of emotion regulation (Gross & John, 1998), the current findings support the proposal that an antecedent-focused strategy in the form of cognitive reappraisal might be more important in moderating suicidal behavior than a response-focused strategy such as expressive suppression that occurs later in the emotion generation process. Previous research has shown that more frequent use of expressive suppression is related to increased SI and suicide desire (Campbell-Sills et al., 2006a, Campbell-Sills, Barlow, Brown, & Hofmann, 2006b; Forkmann et al., 2014; Garnefski & Kraaij, 2006; Gross & John, 2003; Moore et al., 2008); however, the current results show no association between the use of expressive suppression and suicidal behavior. This may be due to the population of the current work. The majority of past studies were conducted using European and American participants (Butler et al., 2007; Forkmann et al., 2014; Soto et al., 2011) and the impact of expressive suppression has been shown to vary across different cultures. For instance, Butler et al. (2007) observed that while emotional suppression was associated with increased negative emotions and hostile behaviors in European Americans, suppression reduced or reversed negativity and hostility in Asian Americans who held Asian values. Consistent with this, Soto et al. (2011) conducted a cross sectional study with 71 European American students and 100 Chinese students from Hong Kong. All participants provided self-report measures of expressive suppression, life satisfaction, and depressed mood and results showed that expressive suppression was associated with adverse psychological functioning for European American participants but not for Chinese participants. Indeed, suppression might be as effective as, or even more effective than, acceptance in regulating negative emotion in Chinese individuals. It should however be noted that this argument is based on findings from individuals reporting relatively mild levels of suicidal behavior. The benefits of suppression in this sample may not extend to individuals reporting more severe symptoms.

The current findings highlight the importance of the target population when investigating suicidal behavior and identifying suicide risk and they show that cultural background is a critical factor in understanding the relationship between emotion regulation and psychological functioning. To account for how emotional expression is moderated by differences in cultural norms, previous studies have shown that western cultures are characterized by individualistic cultural values encouraging free emotional expression (Butler, Lee, & Gross, 2009; Soto, Levenson, & Ebling, 2005; Yuan et al., 2014). For example, compared to Westerners, East Asians generally consider themselves as belonging to a collectivism-oriented, interdependent or low individualism culture (Hofstede, 1994; Kuo, 2011; Nisbett, Peng, Choi, & Norenzayan, 2001). Hofstede (2008) measured the level of individualism across 65 countries including the UK and Hong Kong and reported that the UK was rated higher in terms of individualism compared to Hong Kong.

The results from the current study showed that increased suicidal behavior (SI, past suicide attempts, possible future engagement in suicide) was related to increased avoidance coping and reduced levels of accommodation and self-help coping (however, accommodation and self-help coping did not predict suicidal behavior). Avoidance coping is considered to be a maladaptive coping strategy (John & Gross, 2004; Zuckerman & Gagne, 2003) and the current findings support this. If individuals choose to use avoidance coping to avoid unwanted thoughts and negative emotions (rather than trying to alter them), these emotions may still be readily available despite the effort to keep them out of awareness. The negative thoughts and feelings that are avoided may therefore persist to a greater extent because they have not been resolved (Najmi, Wegner, & Nock, 2007).

Bijttebier and Vertommen (1999) have argued for the importance of investigating individual differences that may contribute to the use of maladaptive coping, such as those relating to personality traits and/or psychopathology. They studied the coping strategies of psychiatric inpatients with personality disorders, grouping coping into three factors of problem solving, seeking social support, and avoidance (using the Coping Strategy Indicator of Amirkhan, 1990). The findings showed a relationship between personality disorders and avoidance coping, particularly for individuals with avoidant or borderline personality disorder. It may be argued that the increased use of avoidance coping may make individuals with avoidant-type personality disorders more at risk of engaging in suicidal behaviors. Related to this, Klonsky, Oltmanns, and Turkheimer (2003) explored self-harm in a large group of military recruits and found those who engaged in deliberate self-harm reported more traits associated with personality disorders (although not specifically avoidant personality disorder). If certain personality traits are associated with maladaptive coping strategies such as avoidance coping, and if these increase the risk of suicidal behaviors, it would be prudent to take account of them when trying to identify those at risk.

In the current study, increased use of accommodation and self-help coping was significantly associated with a reduced risk of suicidal behavior. This suggests that individuals who seek support and express emotion (self-help coping) or accept a problem and positively reframe a situation (accommodation coping) are more likely to be protected against suicidal behavior. While three of the coping strategies (accommodation, self-help, and avoidance coping) proposed by Zuckerman and Gagne (2003) were associated with a risk of suicidal behavior, self-punishment and approach coping showed no relationship with scores on the SBQ-R. This conflicts with previous research showing that suicidal behavior is best predicted by reduced use of approach coping (Pienaar et al., 2007) and increased use of self-punishment coping (Pietrzak et al., 2011). Again, one reason for the disparity in the findings is that the samples used across the different studies vary quite substantially and the individuals within each study may be exposed to different stressors. For example, Pienaar et al.’s (2007) study recruited uniformed police officers from South Africa and Pietrzak et al. (2011) studied veterans in the US. Coping strategies and behavior of individuals from those groups may not be applicable to students who tend to be younger and have less experience in mastering their approach coping (this requires problem solving based on knowledge and past experiences). The use of different coping strategies with regard to suicidal behavior has not been extensively investigated among university students and the present study has therefore shown that it is important to take account of the population and the stressors they may experience before trying to encourage specific coping strategies. This does however highlight a limitation of the current work as it did not consider specific negative life events that may have influenced coping and emotion regulation strategies. The study also did not account for problem-solving ability, or affective disorders that are known to impact suicidal behavior (Chen, 2016; Wagner & Zimmerman, 2006). A further limitation to the current work was the use of a convenience sample, and consequently, the findings are only representative of this group and may not reflect the wider student population.

Comparisons to studies that use a similar population highlight the drawbacks of the current research with regard to both the size and the representativeness of the sample. The students in this study showed a very high level of suicidal behavior, with over 40% classified as having a significant risk of suicidal behavior (based on the criteria of the SBQ-R) and 4.24% reporting a past suicide attempt. A meta-analysis by Mortier, Cuijpers, et al. (2018b) assessed lifetime prevalence of “suicidal thoughts and behaviors” across a number of studies conducted using college students (with a total of 634,662 participants) and found a prevalence of 22.32% for SI and 3.22% for suicide attempts. It should be noted that suicidal behavior was separated into thoughts (SI) and behaviors, whereas in the current study, participants were given a single score for suicidal behavior that incorporated SI. Indeed, research in this field often uses different measures of recording suicidal behavior; therefore, it is difficult to make comparisons, yet the disparity between this study and previous work is a concern. The prevalence of suicide in the current sample is much higher, and it is unlikely this can be attributed to cultural differences as a meta-analysis focusing on Chinese college students found that the prevalence of SI in studies conducted between 2004 and 2013 (including 160,339 participants) was 10.72% (Li et al., 2014). There are many factors that can affect suicidal behavior such as depression, family circumstances, gender, financial situation, and physical health, and the present study did not account for these. This was not the aim of the research but the limitations of the sample should be acknowledged when considering the results.

Overall, the findings show that lower levels of avoidance coping, in combination with higher levels of cognitive reappraisal may explain the resilience of some individuals against suicidal behavior. This was found in a group of undergraduate students reporting relatively mild symptoms of suicidal behavior and therefore the results may be used to improve identification and prevention of suicide at an early stage. A further important point raised by the research is that the use of coping and emotional regulation may be influenced by cultural context and the psychological processes that may be considered risk factors in some populations may be beneficial in others. The research therefore raises the importance of accounting for the background and situation of the individual when trying to identify risk factors to suicide.

## Article Notes

### Author Biographies

**Elsie Ong**, PhD, is a senior research assistant at the University of Hong Kong. Her research focuses on the neurological and cognitive aspects of suicide behavior.

**Catherine Thompson**, PhD, is a lecturer in cognitive psychology at the University of Salford in the UK. Her interests focus on aspects of visual attention, in particular, the top-down control of attention. Her research explores the importance of attentional control in performance and well-being.

### Author’s note

Elsie Ong is now affiliated to Faculty of Education, University of Hong Kong, Hong Kong.
